# Indole-3-Acetic Acid-Producing Yeasts in the Phyllosphere of the Carnivorous Plant *Drosera indica* L

**DOI:** 10.1371/journal.pone.0114196

**Published:** 2014-12-02

**Authors:** Pei-Feng Sun, Wei-Ta Fang, Li-Ying Shin, Jyuan-Yu Wei, Shih-Feng Fu, Jui-Yu Chou

**Affiliations:** 1 Department of Biology, National Changhua University of Education, Changhua 500, Taiwan, R.O.C; 2 Graduate Institute of Environmental Education, National Taiwan Normal University, Taipei 116, Taiwan, R.O.C; Graz University of Technology (TU Graz), Austria

## Abstract

Yeasts are widely distributed in nature and exist in association with other microorganisms as normal inhabitants of soil, vegetation, and aqueous environments. In this study, 12 yeast strains were enriched and isolated from leaf samples of the carnivorous plant *Drosera indica* L., which is currently threatened because of restricted habitats and use in herbal industries. According to similarities in large subunit and small subunit ribosomal RNA gene sequences, we identified 2 yeast species in 2 genera of the phylum Ascomycota, and 5 yeast species in 5 genera of the phylum Basidiomycota. All of the isolated yeasts produced indole-3-acetic acid (IAA) when cultivated in YPD broth supplemented with 0.1% L-tryptophan. Growth conditions, such as the pH and temperature of the medium, influenced yeast IAA production. Our results also suggested the existence of a tryptophan-independent IAA biosynthetic pathway. We evaluated the effects of various concentrations of exogenous IAA on yeast growth and observed that IAA produced by wild yeasts modifies auxin-inducible gene expression in *Arabidopsis*. Our data suggest that yeasts can promote plant growth and support ongoing prospecting of yeast strains for inclusion into biofertilizer for sustainable agriculture.

## Introduction

Plants are populated by microorganisms below and above ground. Above-ground plant parts are typically colonized by microorganisms, such as different genera of bacteria, filamentous fungi, yeasts, algae, and, less frequently, protozoa and nematodes [1]. Microbial species can be isolated from plant tissues and recovered from the surfaces of healthy plants. The aerial habitat colonized by microbes is the phyllosphere. Growth of microorganisms in the phyllosphere is dependent on nutrients from plant metabolites that are secreted to the plant surface, or on compounds from external sources that contact the plant surface. The availability of carbon-containing nutrients on leaves is a major determinant of epiphytic colonization. Simple sugars such as glucose, fructose, and sucrose are the dominant carbon sources in plants and leach from the interior of the plant [2]. However, the leaf surface is a hostile environment for colonists, and is exposed to fluctuating temperatures and relative humidity, as well as repeated changes in the availability of moisture from rain and dew. Stomata, veins, and surface appendages, including trichomes and hydathodes, can all affect nutrient availability in the phyllosphere [3], [4].

The auxin indole-3-acetic acid (IAA) stimulates rapid and long-term responses in plants [5]–[7], and has been identified in plant-associated bacteria [8], [9], fungi [10]–[12], and yeasts [13]–[15]. The role of microbial IAA in plant-microbe interactions has recently received increased attention. Bacteria [9], [16]–[18], fungi [19], and yeasts [20] can all promote plant growth; therefore, IAA-producing microbes have been suggested as sources of biofertilizer [21], [22]. IAA is a signaling molecule in certain microorganisms and modifies gene expression [23], [24]. Therefore, IAA might act as a reciprocal signaling molecule in microbe-plant interactions.

*Drosera indica* L. is a carnivorous plant and a sundew native of tropical countries worldwide. It is distributed from Australia and Asia to Africa, but does not exist in the neotropics. *D. indica* grows in poor, sandy, acidic, and swampy soils. Young *D. indica* plants stand upright, whereas older plants form scrambling stems with only the newest stems growing upright. The narrow leaves of *D. indica* are yellow-green to maroon, alternately arranged, and fringed with gland-tipped tentacles. The tips of the tentacles are formed by sparkling dots of sticky liquid, which trap insects. The plant secretes several enzymes to dissolve a trapped insect and free the nutrients contained within. This nutrient mixture is then absorbed through leaf surfaces to be used by the plant, and can also be used by microbes associated with the plant. *D. indica* is currently threatened because of restricted habitats and collection from the wild for use in herbal industries [25]. Its natural habitat is affected by invasive species, climatic changes, urbanization, and agricultural pollutants. Although *D. indica* is classified as vulnerable, it can also be considered a potentially endangered species because government environmental regulating agencies have enforced stringent conservation measures [26], [27].

Plant-associated microorganisms fulfill valuable functions in plant growth and health. However, no study has reported yeast flora in the phyllosphere of *Drosera* species. Therefore, the purpose of this study was to isolate and identify yeasts in the phyllosphere of *D. indica*, and to evaluate the IAA-producing capabilities of the identified yeasts.

## Materials and Methods

### Sample collection

Green and undamaged *D. indica* plant leaves were collected from Xinfeng Township which is located on the north point of Hsinchu County, Taiwan (N24°52′802″, E120°58′935″). There is no specific permissions were required for these locations/activities. The field studies did not involve endangered or protected species. The annual average temperature of the collection site is around 32°C. The soil temperature is around 26°C and the soil pH is from 6.2 to 6.9. Samples were placed in plastic bags, which were sealed and transferred by an icebox and sent to the laboratory. The samples were maintained at a low temperature (4°C) until yeast isolation procedures.

### Yeast isolation

Yeast was isolated using an enrichment technique by malt extract medium (30 g/L malt extract, 5 g/L peptone) supplemented with approximately 2–3 mL of 100% lactic acid. The *D. indica* leaves were placed into 15-mL test tubes and incubated on a rotary shaker at 30°C for 2 d. A loopful of the enriched culture was streaked onto malt extract agar supplemented with approximately 2–3 mL of 100% lactic acid. Yeast colonies of different morphologies were selected and purified by cross-streaking on malt extract agar. Purified yeast strains were suspended in YPD medium (1% yeast extract, 2% peptone, 2% dextrose) supplemented with 15% v/v glycerol and maintained at −80°C.

### Extraction of yeast genomic deoxyribonucleic acid

Young yeast cultures (1 mL) were transferred to a 1.5-mL tube and centrifuged at 13 000–16 000 *g* for 1 min. The supernatant was discarded and the cell pellet was suspended in 200 µL of lysis buffer (2% Triton X-100, 1% sodium dodecyl sulfate, 100 mM sodium chloride, 10 mM Tris (pH 8.0), 1 mM ethylenediaminetetraacetic acid), to which 200 µL of phenol-chloroform-isoamyl alcohol (25∶24∶1; isoamyl alcohol is optional) and 0.3 g of acid-washed glass beads (0.45–0.52 mm) were added and mixed gently. The samples were vortexed for 5 min to disrupt cells, and then centrifuged at 13 000–16 000 *g* for 5 min. The aqueous layer of each sample was then transferred to a clean tube and 400 µL of 95% ethanol and 16 µL of 3M sodium acetate (pH 5.2) were added. The samples were mixed by inversion and centrifuged at 13 000–16 000 *g* for 5 min. The pellets were washed with 300 µL of 70% ethanol, and the samples were centrifuged at 13 000–16 000 *g* for 2 min before the supernatant was discarded. Ethanol was aspirated with air for 30 min to dry the pellets. Finally, genomic deoxyribonucleic acid (DNA) from each sample was suspended in 100 µL of Tris-EDTA buffer (pH 8.0).

### Yeast identification

Sequences of the D1/D2 domain of large subunit (LSU) and small subunit (SSU) ribosomal RNA (rRNA) were determined from polymerase chain reaction (PCR) products from the genomic DNA extracted from yeast cells. The D1/D2 domain of LSU rRNA was amplified using a PCR with the universal primers ITS-1 (5′-TCCGTAGGTGAACCTGCG-3′) and NL-4 (5′-GGTCCGTGTTTCAAGACGG-3′) [28]. The D1/D2 domain of SSU rRNA was amplified using a PCR and SR1R (5′-TACCTGGTTGATYCTGCC-3′) [29] and BMB-C (5′-ACGGGCGGTGTGTRC-3′) [30] primers. Samples were sent to Tri-I Biotech, Inc for DNA sequencing. A BLAST search of nucleotide sequences was conducted through the National Center for Biotechnology Information homepage (http://www.ncbi.nlm.nih.gov). Yeast identification was accorded to a guideline of Kurtzman and Robnett [31] reported that yeast strains with 0–3 nucleotide differences are conspecific or sister species. And different species were identified if they had>6 nucleotide substitutions.

### Quantification of indole-3-acetic acid by using Salkowski reagent

To quantify IAA produced, yeast isolates were grown in a test tube in YPD medium with or without 0.1% (w/v) L-tryptophan (L-Trp) and incubated in the dark on a shaker at 30°C and 150 revolutions/min (rpm) for 5 d. One milliliter of the cells was pelleted by centrifuging at 3000 *g* for 5 min, and 0.5 mL of the supernatant was mixed with 0.5 mL of Salkowski reagent (2 mL of 0.5M iron(III) chloride and 98 mL of 35% perchloric acid) [32]. After 30 min, color development (red) was quantified using a spectrophotometer (Unico 1200-Spectrophotometer, USA) at 530 nm. A calibration curve using pure IAA was established for calculating IAA concentration. The effects of pH and temperature on IAA production were determined by inoculating YPD medium containing 0.1% (w/v) L-Trp with each yeast isolate and incubating in the dark at pH 4.0, 6.5, or 9.0, or at 37°C, 28°C, or 16°C, on a shaker for 5 d. After incubation, the IAA produced was quantified.

### Effects of exogenous indole-3-acetic acid on yeast growth

To determine the possible biological role of IAA in yeast, the effects of exogenous IAA on the growth of the tested yeasts were investigated by adding various IAA concentrations (0, 312.5, 625, 1250, 2500, 5000 µM) to the YPD medium. Yeast growth was monitored 12 h after IAA treatment by using a spectrophotometer to measure optical density at 600 nm.

### Plant material and growth conditions

The transgenic line *DR5::uidA* of *Arabidopsis thaliana* Col-0 [33] was used to characterize auxin activity in planta. Seeds were surface-sterilized using 5% (v/v) sodium hypochlorite solution with a few drops of Tween 20 for 10 min, and washed 4 times with sterilized water. They were then sown on quarter-strength Murashige-Skoog (MS) medium (M5519, Sigma, MO, USA) supplemented with 1.0% (w/v) sucrose (pH 5.7) and 0.05% (w/v) 2-morpholinoethanesulfonic acid monohydrate, and solidified with 1.5% (w/v) Bacto-agar. Two weeks postgermination, 6 healthy seedlings were randomly selected and inoculated with the supernatants from 5-d cultures (YPD medium with 0.1% L-Trp). A control group was inoculated with the medium only. After an additional 2 d, *DR5*::*uidA* seedlings were stained for GUS activity and cleared to evaluate changes in GUS expression. The plants were then placed in a plant growth chamber with a photoperiod of 16 h light and 8 h dark at 24°C.

### Co-cultivation of *Arabidopsis* plants with yeasts

Yeasts were evaluated *in vitro* for their plant growth-promoting ability using the *Arabidopsis* Col-0 ecotype. Yeasts were inoculated at the opposite ends of agar plates containing 9-d-old germinated *Arabidopsis* seedlings (10 seedlings per plate). Plates were placed vertically in a growth chamber at 25°C with long-day condition (16/8 hr). At 7 d post inoculation, the number of emerged lateral roots was quantified under a dissecting microscope.

### Histochemical analysis

For histochemical analysis of GUS activity, *Arabidopsis* seedlings were fixed with 4% paraformaldehyde in ice-cold 0.1M phosphate buffer (pH 7.0) for 30 min. The samples were incubated on ice and washed 3 times with phosphate buffer (10 min each wash). *Arabidopsis* seedlings were vacuum-infiltrated and incubated overnight at 37°C in a GUS reaction buffer (1 mM 5-bromo-4-chloro-3-indolyl β-D-glucuronide, 5 mM potassium ferricyanide, 5 mM potassium ferrocyanide, and 0.1% Triton X-100 in 100 mM sodium phosphate buffer, pH 7.0) [34]. The stained seedlings were washed 4 times with 70% (v/v) ethanol to stop the reaction and remove chlorophyll. For each treatment, at least 6 seedlings were analyzed. A representative plant was selected and photographed using a stereomicroscope.

### Statistical analysis

Data are expressed as mean ± standard deviation (SD). The significance of differences between groups was determined using Student *t* tests and analyses of variance. *P*<0.05 was considered statistically significant. *P<0.05; **P<0.01.

## Results

### Yeast identification

The D1/D2 domain of the LSU and SSU rRNA gene sequences indicated that we isolated 12 yeast strains in the phyllosphere of *D. indica*. We considered isolates with>6 nucleotide substitutions as different species. Thus, we identified the 12 yeast strains as 7 species in 7 genera (Table 1). Two species were in 2 genera of the phylum Ascomycota: *Aureobasidium pullulans* and one undescribed *Candida* species. Five species were in 5 genera of the phylum Basidiomycota: *Cryptococcus flavus*, *Hannaella coprosmaensis*, *Pseudozyma aphidis*, *Sporisorium reilianum*, and *Ustilago esculenta*. Our results indicated that 16.67% of the isolated strains were ascomycetous yeasts and 83.33% were basidiomycetous yeasts. We identified 5 strains of *Cryptococcus flavus* and 2 strains of *Pseudozyma aphidis* that showed nucleotide sequence divergence within the same species but <6 nucleotide substitutions.

**Table 1:** Yeasts isolated from the phyllosphere of *Drosera indica* L. with the accession numbers of large subunit and small subunit regions (ND = not determined).

strains	closest species (GenBank accession no.) (LSU/SSU)	nucleotide substitutions/total nt (LSU/SSU)		species	accession no. (LSU/SSU)	
**Phylum Ascomycota**						
YL-11	*Aureobasidium pullulans* (FN428878)	5/692	ND	*Aureobasidium pullulans*	KJ917967	
JYC072	*Candida apicola* strain (EU926480)/*Candida floricola* (AB018143)	53/697	14/733	*Candida* sp.	KJ917968	KJ917979
**Phylum Basidiomycota**						
YL-2	*Cryptococcus flavus* (FN428891)	0/691	ND	*Cryptococcus flavus*	KJ917971	
JYC071	*Cryptococcus flavus* (FN428942)	1/697	ND	*Cryptococcus flavus*	KJ917969	
YL-3	*Cryptococcus flavus* (FN428891)/*Cryptococcus flavus* (AB032629)	10/691	758/759	*Cryptococcus flavus*	KJ917972	KJ917981
YL-12	*Cryptococcus flavus* (FN428891)/*Cryptococcus flavus* (AB032629)	11/692	750/751	*Cryptococcus flavus*	KJ917973	KJ917982
JYC073	*Cryptococcus flavus* (FN428942)/*Cryptococcus flavus* (AB032629)	18/750	2/714	*Cryptococcus flavus*	KJ917970	KJ917980
YL-10	*Hannaella coprosmaensis* (FN428945)	0/691	ND	*Hannaella coprosmaensis*	KJ917974	
YL-8	*Pseudozyma aphidis* (FN424100)	2/670	ND	*Pseudozyma aphidis*	KJ917975	
YL-16	*Pseudozyma aphidis* (FN424100)	2/742	ND	*Pseudozyma aphidis*	KJ917976	
YL-9	*Sporisorium cruentum* (AY740156)/*Sporisorium reilianum* (FQ311431)	18/683	0/710	*Sporisorium reilianum*	KJ917977	KJ917983
JYC070	*Ustilago alcornii* (AY740165)/*Ustilago esculenta* (FJ825142)	16/758	0/739	*Ustilago esculenta*	KJ917978	KJ917984

### Production of indole-3-acetic acid by yeast

**Figure 1:**
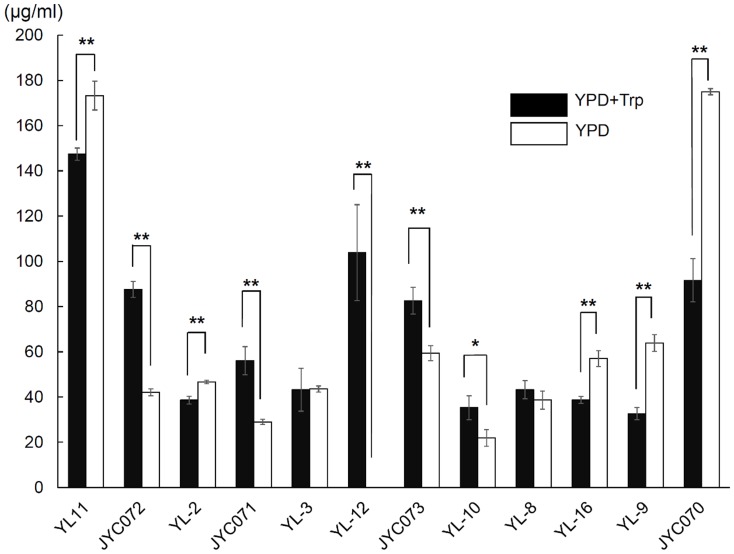
Indole-3-acetic acid production in YPD medium, with or without 0.1% (w/v) L-tryptophan, incubated on a shaker at 30°C and 150 rpm for 5 d. Black bars indicate yeasts cultured in medium with L-tryptophan; white bars indicate yeasts cultured in medium without L-tryptophan.

When we added Salkowski reagent to the yeast species isolates, we observed a color change (to red), indicating that all of the yeasts produced IAA when cultivated in YPD broth supplemented with 0.1% L-Trp (pH 6.5) (Figure 1). IAA production ranged from 32.6 (± 2.7) to 147.4 (± 2.7) µg/mL. *A. pullulans* produced relatively high IAA concentrations (147.4±2.7 µg/mL), whereas *S. reilianum* produced low IAA concentrations (32.6±2.7 µg/mL). Although all 5 *C. flavus* strains produced IAA, the IAA concentration produced was strain-dependent, ranging from 38.6±1.7 to 103.9±21.2 µg/mL (Figure 1). The 2 *P. aphidis* strains exhibited marginal differences in IAA-producing capabilities.

### Yeasts produce indole-3-acetic acid in the absence of exogenous tryptophan

IAA was the first plant hormone discovered; however, its biosynthetic pathway, at a genetic level, remains unclear. IAA biosynthesis in fungi is not well described. Previous studies have suggested that 4 IAA biosynthesis pathways exist in plants and bacteria [35], [36]. In fungi, IAA has been proposed as a metabolite of Trp [37], which was confirmed in later studies in certain species [38], [39]. Rao et al. [40] proposed that a Trp-independent pathway for IAA synthesis exists in baking yeast (*Saccharomyces cerevisiae*). To confirm the existence of a Trp-independent pathway in our tested yeasts, we analyzed IAA production in yeast cultures without Trp. We observed that all but one of our evaluated yeast isolates produced IAA in the absence of exogenous Trp (Figure 1). Five isolates produced higher amounts of IAA, and 5 isolates produced lower amounts of IAA, in the absence of exogenous Trp. Two isolates produced similar amounts of IAA in the presence and absence of Trp (Figure 1). One *C. flavus* strain did not produce IAA in the absence of Trp.

### Influence of pH and temperature on indole-3-acetic acid production

To investigate the effects of environmental changes on IAA production, we adjusted the pH and temperature of the growth medium (containing Trp). Compared with in YPD broth supplemented with 0.1% L-Trp at pH 6.5, in an acidic environment (pH 4.0), 6 isolates produced lower amounts of IAA, 5 isolates produced similar amounts of IAA, and *Candida* sp. produced higher amounts of IAA (Table 2). These results indicated that IAA production is influenced by the pH of the medium. In the majority of yeast species, growth is optimal in a neutral or acidic pH environment. A minority of species thrive in an alkaline environment. When we evaluated IAA production in an alkaline environment (pH 9) we observed that some yeast species thrived; however, none produced IAA.

**Table 2:** Indole-3-acetic acid production in various environments (µg/mL) compared with in YPD broth supplemented with 0.1% L-tryptophan at pH 6.5.

	YPD with Trp (pH 6.5, 28°C)	YPD w/o Trp (pH 6.5, 28°C)	pH 4.0	pH 9.0	37°C	16°C
**Phylum Ascomycota**						
*Aureobasidium pullulans* (YL-11)	147.4(±2.7)	173.3(±6.4)**	33.6(±5.7)**	NG	NG	17.3 (±1.3)**
*Candida* sp. (JYC072)	87.6(±3.6)	42.1(±1.5)**	174.1(±12.8)**	NG	88.9(±6.9)	58.1(±2.0)**
**Phylum Basidiomycota**						
*Cryptococcus flavus* (YL-2)	38.6(±1.7)	46.6(±0.8)**	45.9(±6.8)	NG	NG	11.3(±2.8)**
*Cryptococcus flavus* (JYC071)	56.1(±6.3)	29.0(±1.2)**	31.6(±0.9)*	NG	NG	13.8(±4.5)**
*Cryptococcus flavus* (YL-3)	43.2(±9.5)	43.5(±1.4)	51.3(±2.5)	NG	NG	8.0(±0.4)**
*Cryptococcus flavus* (YL-12)	103.9(±21.2)	0**	39.1(±4.2)**	NG	NG	8.7(±0.6)**
*Cryptococcus flavus* (JYC073)	82.6(±5.9)	59.4(±3.3)**	19.6(±9.5)**	NG	NG	7.4(±1.1)**
*Hannaella coprosmaensis* (YL-10)	35.3(±5.3)	21.9(±3.7)*	4.3(±1.3)**	NG	NG	19.9(±1.5)**
*Pseudozyma aphidis* (YL-8)	43.2(±4.0)	38.7(±4.0)	22.2(±3.6)**	0**	117.3(±11.5)**	62.0(±10.7)*
*Pseudozyma aphidis* (YL-16)	38.7(±1.5)	57.0(±3.5)**	33.9(±7.6)	0**	81.3(±12)**	35.6(±0.4)*
*Sporisorium reilianum* (YL-9)	32.6(±2.7)	63.9(±3.7)**	30.7(±2.3)	NG	37.3(±9.8)	17.5(±0.5)**
*Ustilago esculenta* (JYC070)	91.6(±9.5)	175.0(±1.4)**	93.2(±36.3)	0**	192.6(±38.2)**	563.8(±34.8)**

Data are expressed as mean ± SD. NG = no growth. *P*<0.05 was considered significant.

*P<0.05;

**P<0.01.

When we investigated the effects of incubation temperature on IAA production, we observed that at a high temperature (37°C), only *Candida* sp., *P. aphidis* strains YL-8 and YL-16, *S. reilianum*, and *U. esculenta* thrived. Three of the isolates produced higher amounts of IAA, and 2 isolates produced similar amounts of IAA, at 37°C compared with at 28°C (Table 2). At a low temperature (16°C), all of the isolates thrived. Ten isolates produced lower amounts of IAA, and 2 isolates produced higher amounts of IAA, at 16°C compared with at 28°C (Table 2).

### Effects of exogenous indole-3-acetic acid on yeast growth

**Figure 2:**
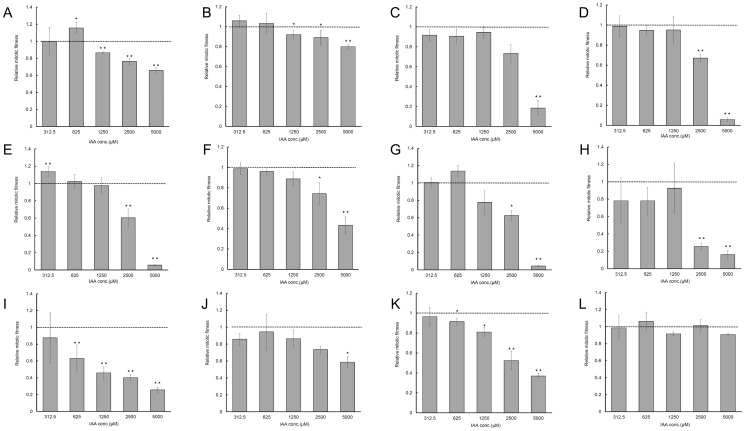
Effects of exogenous indole-3-acetic acid on yeast growth. Yeasts were grown in YPD medium containing different concentrations of indole-3-acetic acid (0–5000 µM). Data are presented as relative growth rates. YPD medium without exogenous indole-3-acetic acid served as a control. (A) *Aureobasidium pullulans*, strain YL-11; (B) *Candida* sp., strain JYC072; (C) *Cryptococcus flavus*, strain YL-2; (D) *C. flavus*, strain JYC071; (E) *C. flavus*, strain YL-3; (F) *C. flavus*, strain YL-12; (G) *C. flavus*, strain JYC073; (H) *Hannaella coprosmaensis*, strain YL-10; (I) *Pseudozyma aphidis*, strain YL-8; (J) *P. aphidis*, strain YL-16; (K) *Sporisorium reilianum*, strain YL-9; (L) *Ustilago esculenta*, strain JYC070.

To elucidate the role of IAA produced by yeast, we evaluated the effects of exogenous IAA on yeast growth. We observed that growth of *U*. *esculenta* was not influenced by the IAA concentrations tested, and that a high IAA concentration (5000 µM) significantly inhibited the growth of 11 of the 12 plant-associated yeasts (Figure 2). In the phylum Ascomycota (Figs. 2A–2B), low concentrations of exogenous IAA (312.5–625 µM) promoted or did not influence yeast growth. However, high IAA concentrations (1250–5000 µM) substantially reduced yeast growth. In the phylum Basidiomycota (Figs. 2C–2L), different species, and different strains of the same species, demonstrated differing growth patterns in response to IAA treatment. In *C. flavus*, 312.5 µM IAA promoted growth of strain YL-3 but exerted no effects on the remaining 4 strains. IAA concentrations of 625–1250 µM did not affect the growth of *C. flavus*. However, 2500 µM IAA reduced the growth of all but one of the *C. flavus* strains (Figures 2C–2G).

The 2 *P. aphidis* strains exhibited differing responses to the various IAA concentrations. At all except the lowest concentrations of exogenous IAA tested, IAA inhibited the growth of strain YL-8. However, only the highest IAA concentration (5000 µM) influenced the growth of strain YL-16 (Figs. 2I–2J).

### Yeasts alter root system architecture in *Arabidopsis*

**Figure 3:**
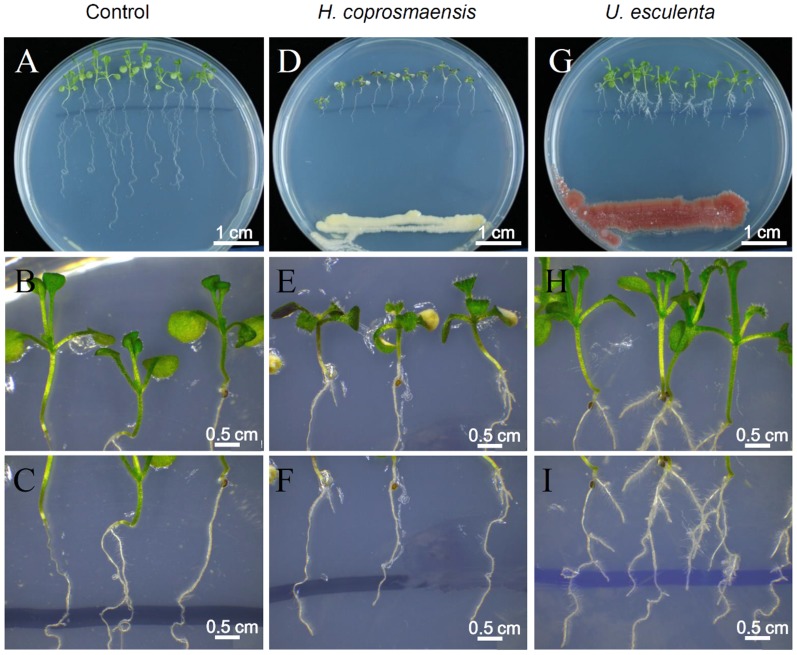
IAA produced by yeasts increased the number of lateral roots and reduced root elongation. (A–C) *Arabidopsis* seedlings (9-d-old) were grown on agar plates containing quarter-strength MS medium. (D–F) The seedlings were inoculated with *Hannaella coprosmaensis* at the opposite ends of agar plates and grown for a further 7 d. (G–I) The *Arabidopsis* seedlings were inoculated with *Ustilago esculenta* at the opposite ends of agar plates, and the seedlings were co-cultivated with the yeast for 7 d.

**Figure 4:**
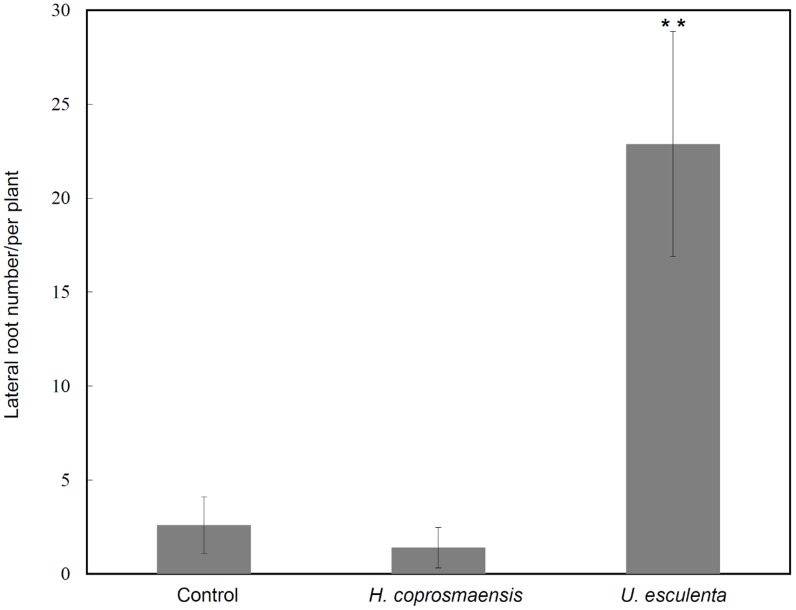
Effects of yeast-producing IAA on *Arabidopsis* lateral roots. Data show mean ± standard deviation (SD) from three groups of 10 seedlings in figure 3. The significance of differences between groups was determined using Student *t* tests and analyses of variance. *P*<0.05 was considered statistically significant. **P<0.01. The experiments were repeated twice with similar results.

**Figure 5:**
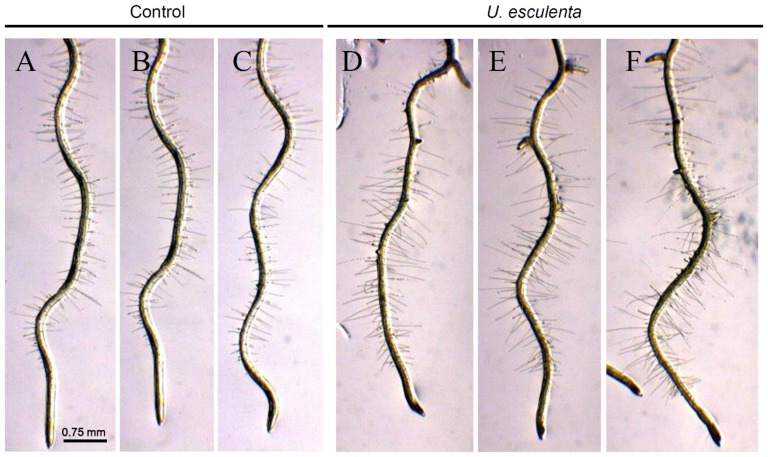
Effects of yeast-producing IAA on root hair formation in *Arabidopsis*. The plants inoculated with *U. esculenta* enhanced formation of root hairs. (A–C) *Arabidopsis* seedlings (9-d-old) were grown on agar plates containing quarter-strength MS medium. (D–F) The seedlings were inoculated with *Ustilago esculenta* at the opposite ends of agar plates, and the seedlings were co-cultivated with the yeast for 7 d.

Lateral root development in *Arabidopsis* has been used as a model for the study of phytohormones signals that regulate postembryonic organogenesis in plants. Development and growth of lateral roots is regulated by phytohormones that coordinate tissue outgrowth in response to environmental changes. The IAA is considered to represent a key regulator of lateral root formation [41], [42]. To get more insight into the effects of IAA produced by yeasts on plant development, the number of lateral roots of *Arabidopsis* seedlings co-cultivated with the yeasts was determined. Yeasts of *U. esculenta* (strain JYC070) exhibited high IAA production in all of the conditions (ex: L-Trp, pH, temperature) and was included in the experiment. In contrast, *H. coprosmaensis* (strain YL-10) produced relatively low IAA in all conditions. As a result, the elongation of primer roots of *Arabidopsis* seedlings was inhibited by co-cultivation with *U. esculenta* or *H. coprosmaensis* (Figures 3A, D, G). However, *Arabidopsis* seedlings co-cultivated with *U. esculenta* led to significant 10-fold increase in lateral root number as compared with those of *H. coprosmaensis* (Figures 3–4). These results indicated that the IAA produced by the yeasts increased the number of lateral roots and inhibited primary root elongation. Subsequently, the effect of IAA produced by the yeasts on the formation of root hairs was analyzed. The *Arabidopsis* seedlings were grown in medium containing *U. esculenta*. At 7 d after co-cultivation with *U. esculenta*, the formation of root hairs was enhanced as compared with the control plants without yeasts (Figure 5).

### Yeasts modify auxin-inducible gene expression in *Arabidopsis*

**Figure 6:**
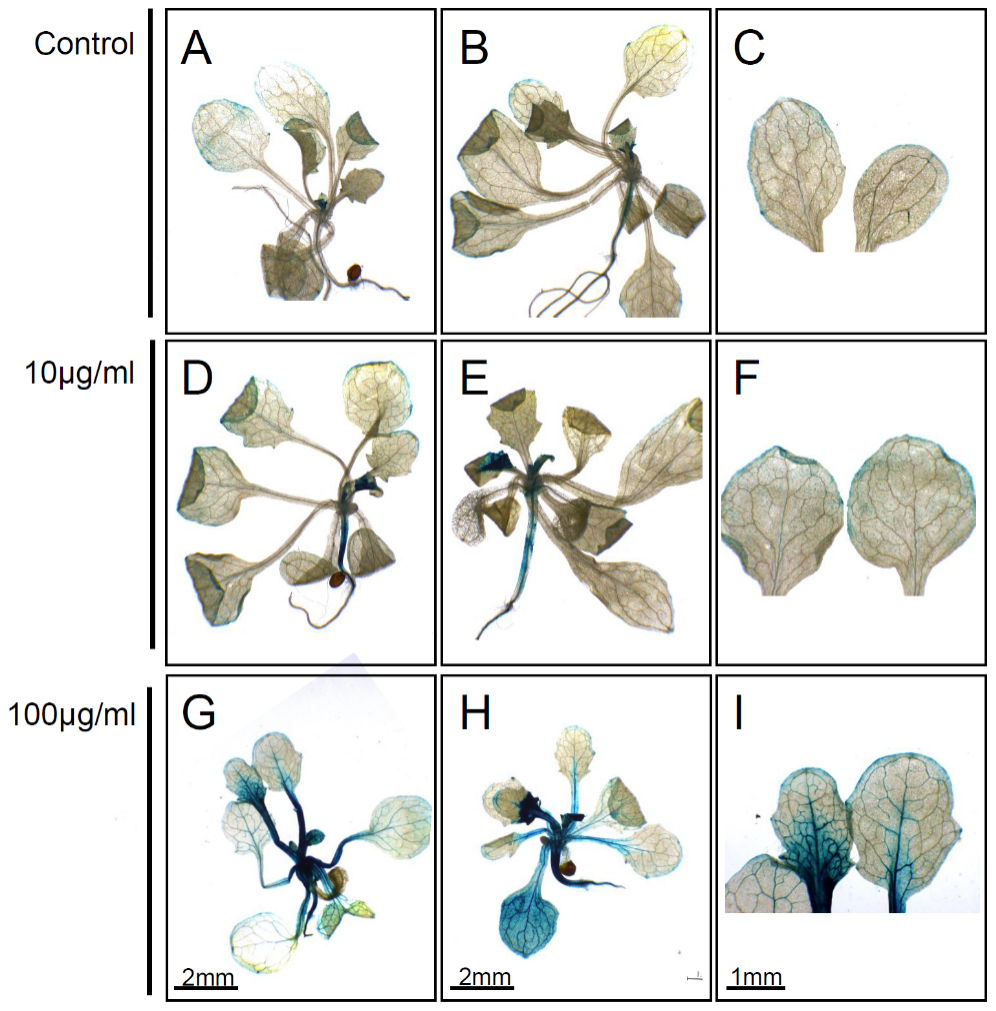
Effects of indole-3-acetic acid produced by *Ustilago esculenta* strain JYC070 on auxin-regulated gene expression. GUS staining of *DR5::uidA Arabidopsis* seedlings grown in the supernatants of 5-d yeast cultures. (A–C) Control group inoculated in YPD medium containing L-tryptophan. In untreated control plants, *DR5*::*uidA* was absent from hypocotyls and young leaves, and was expressed primarily in the leaf margin. (D–F) Seedlings grown in medium supplied with 10 µg/mL IAA produced by *U. esculenta*. (G–I) Seedlings grown in medium supplemented with 100 µg/mL IAA produced by *U. esculenta*. We observed upregulated GUS expression in hypocotyls and young leaves following IAA treatment. Results are obtained from duplicate experiments with similar results. When we treated the transgenic seedlings with the supernatants of other yeasts, we obtained similar results.

The observed effect of yeasts in promoting lateral root development is similar to that described for auxins in plants [43]. Auxin plays a major role in plant growth regulation; however, its role in plant-microbe interactions remains unclear. To investigate whether the IAA produced by yeasts modifies auxin-regulated gene expression in *Arabidopsis*, we inoculated *DR5::uidA* transgenic seedlings with the supernatants from 5-d cultures. The *DR5::uidA* line has previously been used to monitor auxin-regulated gene expression in plants [44]. Figure 3 shows histochemical stains of transgenic *DR5::uidA* seedlings grown in the supernatants of *U. esculenta* (strain JYC070) cultures. In untreated control plants, *DR5*::*uidA* was absent from hypocotyls and young leaves, and was expressed primarily in the leaf margin (Figs. 6A–6C). We observed GUS activity in the hypocotyls and young leaves of *DR5::uidA* seedlings grown in 10 µM IAA produced by *U. esculenta* (Figs. 6D–6I). Although patterns of GUS expression in *DR5::uidA* seedlings treated with 100 µM IAA or 10 µM IAA were similar, GUS expression was higher in the 100 µM IAA-treated seedlings than in the 10 µM IAA-treated seedlings. These results indicated that IAA produced by yeasts upregulates the expression of auxin-inducible gene markers in plants. When we treated the transgenic seedlings with the supernatants of other yeasts, we obtained similar results (data not shown).

## Discussion

According to our research, our study is the first to investigate yeasts in the phyllosphere of the carnivorous plant *D. indica* L., the IAA-producing abilities of such yeasts, and the effects of environmental factors on yeast IAA production. Our results strongly support the existence of a Trp-independent IAA biosynthetic pathway in yeast and suggest the potential use of IAA-producing yeasts as biofertilizer inoculants to promote plant growth.

### Yeasts associated with *D. indica* L

Among the yeast species identified in the phyllosphere of *D. indica*, *A. pullulans* is a common inhabitant of leaves of plants [45]–[48] and has been used as an indicator of environmental pollution [49]. *A. pullulans* is also used for biological control of plant diseases [50]–[52] and has biotechnological applications [53], [54]. *C. flavus* exists on *Sphagnum* mosses and the leaves of vascular plants [55]. Members of the anamorphic genus *Cryptococcus* might represent one of the dominant species in plant phyllospheres [56]–[58]. *P. aphidis* is a heterobasidiomycetous yeast related to smut fungi of the *Ustilago* genus, which secretes extracellular metabolites that inhibit various fungal pathogens, and is nonpathogenic to plants. Thus, *P. aphidis* has potential use as a biocontrol agent of fungal pathogens [59]. *S. reilianum* is a phytopathogenic fungus that produces maize head smut [60], and has caused substantial economic damage in temperate and relatively dry areas where maize is cultivated. *S. reilianum* has been established as a model organism to investigate fungus-host interactions at a molecular level, and its genome sequence was recently published [61]. Further investigation on whether *S. reilianum* causes disease in *D. indica* L. is warranted. Similar to *S. reilianum*, *U. esculenta* is a biotrophic smut fungus that parasitizes *Zizania latifolia*, an edible aquatic vegetable from the southern China region [62]. However, the mycelium of *U. esculenta* exerts minimal pathologic effects on plants tissues, with no signs of chlorosis or necrosis, though it does inhibit flowering. Therefore, the relationship between *U. esculenta* and its host is considered a harmonious interaction [63]. However, it is noticeable that some of the yeasts isolated from the *D. indica* have already been isolated from different plant phyllospheres. Some of them have been shown to be pathogenic, which need to be examined before using its plant growth promoting activities as biofertilizers. It needs more research to investigate the host specificity of these yeasts with plants and the molecular mechanisms of yeasts-plant communication. Our results show the role of IAA in plant-yeast interactions, and supports the potential use of yeasts as plant biofertilizers in controlled and field conditions [64]. However, in addition to phytohormone production (IAA), a diverse range of plant growth promoting characteristics, including pathogen inhibition, ACC (1-Aminocyclopropane-1-Carboxylate) Deaminase, phosphate solubilisation, N and S oxidation, siderophore production and stimulation of mycorrhizal-root colonization should be investigated in future studies before inclusion of these yeasts in the commercial biofertilizer product [64]–[66].

### Production of indole-3-acetic acid by yeast

Our assessments of the IAA-producing capabilities of the phyllosphere yeasts revealed that all of the investigated yeasts produced IAA. Observations that the different strains of one species exhibit different IAA-producing capabilities indicate that IAA production is strain-dependent in yeasts associated with *D. indica*. Previous studies evaluating other microorganisms similarly reported differences in IAA biosynthesis among strains within the same species [67], [68]. *A. pullulans* produced higher amounts of IAA than the other species did. However, a precise analytical method, such as high-performance liquid chromatography or gas chromatography-mass spectrometry, should be used to confirm the IAA concentration produced by *A. pullulans*.

Trp-dependent and Trp-independent IAA biosynthetic pathways reportedly coexist in plants [36], [69] and microbes [8]. However, the majority of previous studies on IAA biosynthesis evaluated Trp-dependent processes. Few studies have evaluated the Trp-independent pathways of IAA biosynthesis. The intermediates, intermediate stages, and genes involved in Trp-independent pathways have yet to be defined. In plants, 4 Trp-dependent pathways have been proposed: the indole-3-acetamide (IAM), indole-3-pyruvic acid (IPA), tryptamine, and indole-3-acetaldoxime pathways [36]. Although different plant species might use specific strategies or modifications to optimize synthetic pathways, plants would be expected to share evolutionarily conserved core mechanisms for IAA biosynthesis. Little is known on the biochemical processes involved in Trp-independent IAA production in plants [70], [71]. Several bacterial IAA biosynthetic pathways might exist in *Azospirillum brasilense*, in which IAA can be synthesized from Trp through IAM, IPA, and indole-3-acetonitrile pathways [72]. However, feeding experiments with labeled precursors have indicated that Trp-independent IAA production in *Az. brasilense* is derived from intermediates in Trp pathways [8], [72].

The fungal IAA biosynthetic pathway has not been widely investigated [10], [40], [73]. In this study, all of the isolated yeasts produced IAA when cultivated in YPD broth supplemented with 0.1% L-Trp. Limtong and Koowadjanakul [13] collected yeasts from the phyllosphere of various plant species in Thailand, observing that approximately 37.7% of the investigated yeast strains produced IAA. Xin et al. [14] isolated 3 endophytic yeasts from *Populus* trees, which all produced IAA when incubated with L-Trp. These studies suggest that IAA production is a common feature in several types of yeast.

Rao et al. [40] observed that *S. cerevisiae* incubated with L-Trp synthesized 4-fold higher amounts of IAA, compared with the wild type, when the genes (*ALD2*, *ALD3*) involved in the final stage of IAA synthesis from Trp were compromised. The amount of IAA produced by the *ald2*Δ*ald3*Δ mutant was similar to that produced by the wild type in the absence of exogenous Trp. In this study, all yeast isolates but one produced IAA in the absence of exogenous Trp, suggesting the existence of an alternate IAA synthesis pathway in yeast. Additional studies are required to detect and quantify the intermediates in this pathway by using advanced analytical techniques and functional genomics. Such studies can increase knowledge on the various IAA biosynthesis pathways in IAA-producing organisms.

### Environmental factors modulating indole-3-acetic acid production

Numerous environmental variables can influence IAA biosynthesis [8], including pH and temperature. Strzelczyk et al. [74] reported that auxin biosynthesis is favored in mycorrhizal fungi at pH 6.0–9.0. Studies have observed similar trends in rot fungus *Pleurotus ostreatus* [75] and *Nectria pterospermi* [76], a pathogenic fungus of the canker of maple-leaved pterospermum. Our results support that IAA production is influenced by the pH of the medium. We observed that 6 of the yeast isolates produced lower amounts of IAA, 5 isolates produced similar amounts of IAA, and one isolate produced higher amounts of IAA in an acidic environment compared with in a nearly neutral environment. All of the isolates were unable to produce IAA in an alkaline environment (pH 9).

When we investigated IAA production by the yeast isolates at different temperatures, we observed that in the majority of the yeasts, 28°C was the optimal temperature for IAA production, compared with 37°C and 16°C. In previous studies, fungal IAA production was also maximal at 28°C [77], [78]. However, 3 of our isolates produced higher amounts of IAA at 37°C, and 2 isolates produced higher amounts of IAA at 16°C, compared with at 28°C (Table 2). Additional analyses are required to determine the influence of factors such as substrate concentration, carbon and nitrogen sources, and precursor (Trp) concentration on IAA biosynthesis mechanisms to facilitate optimizing the culture environment.

### Effects of exogenous indole-3-acetic acid on yeast growth

We evaluated the effects of exogenous IAA on yeast growth to determine the biological role of IAA. IAA is considered a signaling molecule in bacteria and might directly affect bacterial physiology [8]. Studies have indicated that fungi can recognize chemical cues that signal the presence of the plant host to induce invasion. The presence of IAA at wound sites in plants suggests that IAA is an attractant for fungi. Our study results indicated that high IAA concentrations inhibit yeast growth. Previous studies similarly reported that IAA inhibits microbial growth in *Agrobacterium*, several other plant-associated bacteria [79], and fungi [24], [80]. Our results also indicated that low exogenous IAA concentrations promote yeast growth. In previous studies, IAA promoted the growth of *Fusarium delphinoides* [80] and *S. cerevisiae* [24]. IAA can exert stimulatory and inhibitory effects on yeasts, and such effects are strain-dependent. Prusty et al. [24] reported that IAA promoted the growth of filamentous forms of *S. cerevisiae* and promoted invasion, which supports the role of IAA as a signaling molecule that regulates yeast growth. However, additional functional genomic studies are required to fully elucidate the involvement of IAA in microorganism-plant interactions and direct microbial conversion.

### Yeasts alter root system architecture in *Arabidopsis*

Fungal species have been applied to plants for the purpose of growth enhancement, with a positive effect on plant weight, crop yields, and disease control [81]. In maize (*Zea mays*), root growth is markedly enhanced by colonization with *Trichoderma harzianum*. This enhancement can rescue some stress-induced growth reduction and reverse oxidative injury [82]. The ectomycorrhizal fungus *Laccaria bicolor* has also been proved to stimulate lateral root formation in poplar and *Arabidopsis* through auxin transport and signaling [83]. In this study, we found that co-cultivation of *Arabidopsis* plants with the yeasts enhances formation of lateral roots, suggesting that the effects are mediated by auxin (Figs. 3–5). Interestingly, the yeasts did not enhance the overall biomass in the aspect of shoot growth. Instead, co-cultivation of *Arabidopsis* plants with the yeast specifically promoted formation of lateral root and root hairs (Figs. 3–5). Lateral roots extend horizontally from the primary root and to anchor the plant securely into the soil. Root hairs increase the surface area of a root and play critical roles in the uptake of water and nutrients. These results implied a beneficial effect of yeast inoculation on plant growth and development.

### Yeasts modify auxin-inducible gene expression in *Arabidopsis*

Although previous studies have characterized IAA-producing microorganisms [8], [84], the interactions between microorganisms and plants are not well described. We hypothesized that yeasts produce IAA as a colonization strategy. Our hypothesis is supported by Prusty et al. [24], which indicated that IAA induces adhesion and filamentation in *S. cerevisiae*. Contreras-Cornejo et al. [85] similarly proposed that auxin produced by fungi promotes plant interactions by circumventing basal plant defense mechanisms. Navarro et al. [86] identified that inhibition of auxin signaling restricts the growth of *Pseudomonas syringa* in *Arabidopsis*, implicating auxin in plant disease susceptibility. Our results demonstrate that IAA produced by yeasts upregulates the expression of auxin-inducible plant gene markers, which suggests that IAA plays a major role in plant signaling.
